# Nurse leaders’ appraisal of work-organizational interventions during COVID-19: an interview study in German hospitals and long-term care facilities

**DOI:** 10.1186/s12912-026-04781-y

**Published:** 2026-05-21

**Authors:** Maria Zink, Annika Hofmann, Johannes Wendsche, Marlen Melzer

**Affiliations:** 1 Federal Institute for Occupational Safety and Health (BAuA), Fabricestrasse 8, 01099 Dresden, Germany; 2 Technische Universität Dresden (TUD), Dresden, Germany

**Keywords:** COVID-19 pandemic, Healthcare management, Nurse leadership, Nursing staff, Organizational change, Process evaluation, Work environment, Crisis management

## Abstract

**Background:**

The COVID-19 pandemic disrupted healthcare systems globally, requiring rapid organizational changes in inpatient care. Nurse leaders played a central role in adapting and implementing work-organizational interventions under challenging conditions. Understanding factors influencing intervention success is essential for building resilient healthcare systems. This study explored nurse leaders’ perspectives on the success of work-organizational interventions during the COVID-19 pandemic in German hospitals and long-term care facilities. It examined which interventions were perceived as successful, key implementation facilitators and barriers, and how these related to perceived success.

**Methods:**

This study employed an exploratory sequential mixed-methods design. A qualitative, retrospective study using semi-structured interviews was conducted, supplemented by quantitative organizational data. Fifty-three nurse leaders across strategic, middle, and operational management levels in hospitals and long-term care facilities in the German federal state of Saxony participated. Sampling ensured variation in care settings, ownership types, regions, and pandemic impact. Interviews were guided by a framework for evaluating organizational interventions, focusing on perceptions of intervention success, implementation processes, and context. Data was analyzed using qualitative content analysis. In the following, data was transformed into quantitative variables to examine frequencies and statistical associations of variables under investigation.

**Results:**

Nurse leaders perceived 36 out of 51 interventions as successful, particularly those addressing infection control, staff scheduling, and teamwork or interprofessional cooperation. Perceived success was facilitated by effective collaboration, leadership support, and staff motivation. Barriers included staffing shortages, infrastructure issues, and psychological strain. Staff acceptance of interventions showed a statistically significant moderate association with perceived success, while internal initiation, nursing staff participation, active leadership role, systematic evaluation, management hierarchy level, and hospital setting were not statistically significant and showed weak or no association.

**Conclusions:**

Our results show that nursing staff acceptance is critical for successful intervention implementation during crises. Clear communication, participatory decision-making, and contextual flexibility enhance both acceptance and perceived success. These findings offer practical guidance for strengthening healthcare system resilience in ongoing and future crises.

**Supplementary Information:**

The online version contains supplementary material available at 10.1186/s12912-026-04781-y.

## Social media abstract

Nurse leaders report staff acceptance, clear communication, and individual as well as facility flexibility as key to effective pandemic response and future crisis readiness.

**Supplementary Information:**

The online version contains supplementary material available at 10.1186/s12912-026-04781-y.

## Introduction

The COVID-19 pandemic, officially declared by the World Health Organization in March 2020, had a strong impact on the working conditions in healthcare worldwide [1, 2]. Due to the rapid transmission of the severe acute respiratory syndrome coronavirus 2 (SARS-CoV-2) via the respiratory tract, case numbers increased exponentially [3, 4]. This was also accompanied by high rates of seriously ill hospitalized patients [5]. Against this background, healthcare and nursing facilities were compelled to respond rapidly to the evolving pandemic, necessitating extensive organizational changes and the implementation of new measures.

## Background

### Organizational changes and interventions

The need to prevent the further spread of the virus and to simultaneously ensure adequate care for both infected as well as otherwise ill patients led to diverse new requirements for nurses in hospitals and long-term care facilities [6, 7]. For example, to ensure infection control, spatial reorganization to isolate infected patients, the implementation of testing procedures for staff and patients and the obligation to wear personal protective equipment (PPE) have been necessary interventions which changed work organization quickly and significantly [2, 8–10].

Since the virus was novel and mutated quickly, the specific symptoms and transmission paths were still being explored as the pandemic unfolded. Consequently, regulations and recommendations regarding the frequency of testing, or distancing rules changed almost daily during the most critical pandemic waves [8, 10].

### Effects of working conditions and nurses’ health

Several studies in the nursing context found that the frequent and rapid reorganization of routines as well as the reassignment of atypical tasks led to increased work stress for nurses [9, 11–13], higher insecurity and higher work-related strain [8, 10].

In addition to these psychosocial risks nurses were exposed to a considerably higher risk of an infection with SARS-CoV-2 in contrast to other occupational groups [1, 7, 14, 15]. In Germany, where our study was conducted, employees in nursing and healthcare reported the highest number of pandemic-related sick leave days across multiple phases [3, 15].

Additionally, the permanent demand to wear PPE caused several problems. Initially, many hospitals - and especially long-term care facilities - faced a lack of required equipment, which resulted in improper use (e.g., items being worn for too long; [9, 10, 16, 17]). Furthermore, nurses reported heat strain, respiratory problems, skin irritations, and impaired communication with patients and colleagues due to PPE [8, 14, 18].

### Effects of high staff absence

Beyond the health-related consequences of infection, increased absence rates among nursing staff, coupled with a high patient load, led to significant changes in working conditions that also impacted the physical and mental well-being of the remaining nurses [3, 7]. For instance, nurses were often exposed to an increased number of working hours by taking on additional or longer shifts, a reduced number or length of rest breaks, and shortened intershift rest periods [3, 17, 19]. This led to increased work intensity, as long-term care facilities had to manage additional and unfamiliar tasks, while intensive care units faced a higher number of severely ill patients [3, 7, 17]. Nevertheless, repeatedly having to manage the same disease led to reduced task variety, which in turn increased perceived work intensity [3, 20]. This contributed to mental health impairments (e.g., sleep disturbances, anxiety, and depression) as well as increased intentions to leave the organization [3, 7, 11, 14, 19, 21]. At the same time, legal restrictions and stigmatization limited social interactions during leisure time, further harming nurses’ mental health and depleting a vital resource for building resilience [3, 22, 23].

### Improving working conditions and health

Protecting nurses’ mental and physical health in such critical situations must be a top priority [9, 11]. Ensuring that nurses can perform their duties with dedication and without adverse health effects is not only an ethical responsibility but also essential for maintaining a functional healthcare system and effectively managing future pandemics [1, 9, 11].

Appropriately applied interventions can have a significant impact on nurses’ wellbeing and health. Some of these interventions have already been investigated in scientific studies. For instance, Zaghini et al. [24] evaluated the effectiveness of a multi-component, four-month prevention program, including PPE training, increased nurse-to-patient ratios in COVID-19 and intensive care units, and additional meetings to discuss challenging cases. After completing this intervention, the nurses reported improvements in their psychosocial work environment and workplace relationships, increased job control and perceived autonomy, better managerial and peer support, as well as enhanced management and communication of organizational changes. However, the quality of evidence supporting the effectiveness of this intervention was limited, as the study employed an observational design and did not account for potential confounding factors such as the participants’ age.

Accordingly, evaluating effects alone is insufficient to fully assess the effectiveness of an intervention. Factors such as the employees´ perceptions of the intervention activities, the information provided about the intervention purpose(s), and external challenges can affect the success of implementation [25]. Therefore, a process evaluation is crucial. Consequently, it is firstly necessary to find out who initiated the intervention and what strategy or intention guided the intervention implementation. The nurses’ perception of interventions’ objectives and initiators play a crucial role in their willingness to support it [25]. Furthermore, nurses’ participation in the implementation process should be considered, as involvement can empower employees and contribute to positive intervention outcomes [26, 27]. It is also essential to examine nurse leaders’ own engagement. If they actively and supportively participate in the implementation process, it may positively influence nurses’ perception of working conditions and own wellbeing [28]. Additionally, it is necessary to explore which facilitating or hindering factors affected the implementation of interventions [25]. Evaluating these key aspects helps to gain a better understanding of the implemented interventions’ generalizability and validity, and to identify the aspects mainly determine success or failure of an intervention. This in turn forms the basis for developing useful strategies for effective action in future pandemics [25]. Finally, the perspective of nurse leaders is especially valuable as this group of people has deep insights into the intervention implementation process [29].

To contribute to this emerging approach of evaluating the effectiveness of organizational interventions, we conducted qualitative interviews with nurse leaders to identify both successful and less successful interventions from their point of view [30] (Q1). In doing so, we adhered to the process evaluation framework proposed by Nielsen and Randall [25] (Q2) and explored their relevance for participants’ appraisal of intervention success or failure (Q3). The following core research questions will be addressed in the current publication:

Q1: *Which work-organizational interventions of inpatient care facilities (clinics*,* nursing homes) are particularly perceived as successful vs. less successful by nurse leaders?*

Q2: *Which key aspects of the implementation process are described?*

Q3: *Is there an association between the participants’ appraisal of intervention and the four key implementation factors such as nursing staff participation*,* nurse leaders’ roles*,* evaluation and initiation of the intervention*,* as well as the acceptance of the interventions by nursing staff?*

## Method

### Study design

This study employed an exploratory sequential mixed-methods design. Firstly, we conducted retrospective, qualitative semi-structured interviews. Secondly, qualitative data gained by content analysis is transformed into quantitative variables to perform statistical tests. This supplemental quantitative research strategy was intended to complement, rather than replace, the qualitative analysis. More specifically, it was conducted to explore assumptions derived from the process evaluation framework proposed by Nielsen and Randall [25], while adding value by enabling systematic comparison across cases and identifying patterns in the qualitative data.

Nurse leaders from various management levels (e.g., strategic, middle, and operative management) in hospitals and long-term care facilities across Saxony were interviewed. The rationale for recruiting nurse leaders is based on the assumption that successful implementation of new interventions into work processes—particularly crucial during crises—requires consideration of multiple pathways and diverse organizational levels [31, 32]. This concept has been supported by sociotechnical models of analyzing nursing care [25, 31, 33, 34]. It is widely acknowledged that middle managers play a crucial role in implementing work-related changes [25, 28, 29, 34]. A well-educated nurse leader who understands the objectives of new regulations can effectively inform and motivate employees, thereby driving successful change [28, 34]. Since they mediate between the requirements of senior management and the needs of their employees and their patients, they also offer valuable insights into working processes during a pandemic [28, 34, 35]. For these reasons, nurse leaders were chosen as the target group for the interviews.

We developed the interview questions following the suggestions for process evaluation by Nielsen and Randall [25], according to which it is essential to understand the development process of interventions. These considerations about nursing staff participation, nurse leaders’ roles, evaluation and initiation of the intervention, as well as the acceptance of the interventions by nursing staff or hindering and promoting factors formed the basis for the development of the interview guide, which addressed perceptions of both successful and less successful interventions - reported in this study - alongside questions on strategies and organizational interventions implemented during pandemic management, reported in an already published study (for further information see Zink et al. 2025 [36]).The interview data were supplemented by quantitative organizational data (e.g., facility size, number of nursing staff employed) and interviewee socio-demographic details (for the full translated interview guide see Supplementary File 1). The Ethics Committee of the German Federal Institute for Occupational Safety and Health approved this study (research project F2541, 047/2021).

### Recruitment of nurse leaders

A purposive sampling strategy was employed to ensure variation across care settings, regional distribution, levels of management, and types of ownership (private, public, and non-profit). Sample diversity is essential to capture a wide range of pandemic-related experiences, as heterogenous samples strengthen the depth and validity of qualitative findings. To include diverse perspectives, the intention was to recruit at least three nurse leaders per organization: one from strategic or middle management and two from operational management across different departments. To ensure sample diversity across facility and participants, the aim of a minimum of 48 nurse leaders from eight hospitals and eight long-term care facilities in Saxony was defined. Eligibility criteria for participants included holding a leadership position in a Saxon hospital or long-term care facility during the COVID-19 pandemic and having been employed in the same institution throughout that time. Additional criteria were that the participants work in units that were particularly impacted by the pandemic, such as COVID-19 units, ICUs, closed dementia units, or long-term care facilities that experienced outbreaks.

Recruitment was conducted via email and telephone by two members of the research team. A total of 17 hospitals and 19 long-term care facilities were contacted multiple times. Interested institutions received detailed study information, and written informed consent was obtained prior to participation. Eleven hospitals (64% response rate) and seven long-term care facilities (37% response rate) agreed to participate. Declined invitations were mainly due to limited time resources, ongoing staffing shortages, increased patient loads, negative associations with the pandemic, and institutional barriers such as the time needed to complete the organizational survey or obtain internal study approval. One hospital withdrew before data collection due to illness among its leadership team. All 53 participants (31 hospitals, 22 long-term care facilities) received compensation of 50 euros for their participation.

### Data collection

An interview guide was collaboratively developed, revised, and tested both within the research team and with external participants in nursing. Substantial modifications followed the internal piloting phase, which incorporated feedback from a former nurse and a student of psychology. Although the research team discussed repetition of the pilot interviews, they ultimately decided to include them in the final data set due to the high quality of these interviews and to avoid placing excessive burden on the participants. Prior to the main data collection, all interviewers underwent training in applying the interview guide and participated in mutual observation during the pilot phase as well as the first six interviews.

A total of 53 interviews were conducted between July 2022 and June 2023—after the official end of the COVID-19 pandemic. All interviews took place in quiet settings within participants’ workplaces, with only the participant and researcher present to ensure confidentiality and minimize disruptions. The questions regarding the evaluation of the interventions (i.e., successful vs. unsuccessful interventions) were part of a broader interview study on pandemic strategies and interventions in inpatient care. These questions were asked after the participants had responded to more general questions about strategies and interventions, thereby activating their memories of COVID-19 pandemic management and enabling them to recall both successful and unsuccessful interventions in greater detail. The interviewers also took notes during each interview (see first page of Supplementary File 1).

### Data analysis

The 53 audio files were transcribed and analyzed using descriptive qualitative content analysis in MAXQDA following the methodology outlined by Kuckartz and Rädiker [37]. Responses were deductively coded and categorized as either successful or unsuccessful perceived interventions, following the semi-structured interview question. Organizational interventions were defined as planned or reactive, top-down measures that directly modify work content or context, including tasks, resources, the work environment, schedules, or social arrangements. This definition draws on Zink et al. [14].

For each intervention type, one researcher applied deductive coding based on the interview questions. The same researcher then paraphrased the responses, which were subsequently reviewed for accuracy by a second researcher.

Original text and paraphrases for each interview questions were exported to Excel and, in turn, further summarized into distinct themes or categories within each question. These themes and categories were generated by a third researcher and discussed with the second researcher. For transforming the themes and categories into quantitative data, each received a number within its respective quantitative variable. Intervention main category, aim, hindering and promoting factors, challenges and source of challenges as well as idea for intervention were transformed into nominal variables. Initiation of implementation, participation of nursing service, acceptance by nursing staff, role of nurse leader, evaluation, setting and hierarchy-level of participant were transformed into ordinal variables. In a final step, interventions percieved as successful or less successful, along with their respective categories for each question, were imported into SPSS, where quantitative analyses (descriptive statistics, Pearson correlation with two-tailed significance tests for r, *p* < 0.05) were subsequently performed. Transformation was discussed within the research team and was generated by one researcher and checked by a second researcher. A codebook was created, representing each step and ensuring consistency.

Although 53 nurse leaders were interviewed, it became evident during analysis that some interviews lacked sufficient quality regarding the questions on intervention appraisal. Quality and inclusion of interviews were discussed by two researchers. Finally, 36 successful and 15 less successful interventions from nine hospitals and six long-term care facilities were included in the final analysis. Table 1 presents the sample composition regarding included interventions. A high degree of heterogeneity regarding institutional characteristics (region, ownership, size) and participant characteristics (sex, age group, hierarchical level) was achieved. The study design does not permit reliable conclusions about data saturation, as the overall study aim was not specifically focused on the questions analyzed in this paper.

**Table 1:** Facility and participants characteristics for study population of included interventions

Facility	Hospital					Long-term care				
Ownership	Public		Private	Charitable	Total	Public		Private	Charitable	Total
N	4		3	2	9	0		1	5	6
Level of care	level 1	level 2	level 3	specialist hospital	Total	/		/	/	/
N	4	3	0	2	9	/		/	/	/
Regional distribution	metropolitan		non-metropolitan		Total	metropolitan		non-metropolitan	rural	Total
N	5		4		9	1		2	3	6
Size	min	max	M	SD	Total	min	max	M	SD	Total
Nurses	120	2000	666.7	607.3	8	30	102	66.0	32.9	4
Beds	165	1451	506.7	506.6	8	58	134	108.5	34.5	4
Participants										
Management level	strategic		middle	operative	Total	strategic		middle	operative	Total
n (%)	4 (21%)		3 (16%)	12 (63%)	19 (100%)	3 (22%)		5 (36%)	6 (42%)	14 (100%)
*Age group* (in years)	26–35	36–45	46–55	56–65	Total	26–35	36–45	46–55	56–65	Total
n (%)	3 (16%)	8 (42%)	6 (32%)	2 (10%)	19 (100%)	3 (21%)	3 (21%)	3 (21%)	5 (37%)	14 (100%)
Sex	female		male		Total	female		male		Total
n (%)	13 (68%)		6 (32%)		19 (100%)	12 (86%)		2 (14%)		14 (100%)

Note. n = number of participants; N = number of facilities; M = mean; SD = standard deviation

### Quality criteria of qualitative research

According to the Consolidated Criteria for Reporting Qualitative Studies (COREQ; [38]), qualitative studies should address aspects such as the research team, reflexivity, study design, analysis, and findings. To ensure comparability across the interviews, an interview guide was used (Supplementary File 1). This questionnaire was developed in collaboration with the interviewers and finalized after two pilot interviews. To standardize its application across the three interviewers (one male, two females), an interviewer training as well as mutual observations were conducted. All three interviewers had prior experience conducting semi-structured interviews. Multiple researchers discussed the coding system, the categories (two researchers) and the results (three researchers).

## Results

Based on participants’ self-reports, thirty-six of the 51 interventions were perceived as successful regarding the organization of nurses’ work to manage pandemic-related challenges. Fifteen reported interventions were perceived as unsuccessful.

### Question 1: Main themes and interventions

Interventions perceived as successful cover the following eight themes: Interaction with relatives and close associates, work flow, staff scheduling, deployment of experts, infection control, communication and information flow, provision of patient care. Negative interventions focus on these six themes: Staff scheduling, infection control, communication processes and coordination of information flow, team work, training, and internal or external support.

Examples for each theme are given in Table 2. Interventions relating to work flow, staff scheduling, resident-relative-interaction and provision of care were almost exclusively perceived as successful, whereas there are mixed appraisals for interventions regarding communication, team work and infection control. The most frequently mentioned interventions —regardless of their appraisal—related to infection control (see Table 3).

**Table 2:** Examples of reported interventions, categorized by theme and perceived success

Themes	Interventions perceived as successful	Interventions perceived as less successful
Infection control	• Mandatory mask policy • Testing strategy • Establishment of dedicated, interdisciplinary COVID-19 care unit • Cohorting of staff and patients, and spatial separation of residential areas • Procurement of protective equipment • Appointment of hygiene officers • Postponement of surgeries beyond official guidelines in suspected COVID-19 cases	• Testing strategy (e.g., frequency, regarding vaccination status) • Cohorting of staff following an outbreak within the facility • Hygiene measures • Visitation ban
Communication and information	• Adaptation of communication management to ensure rapid, direct information flow between leadership and staff (telephone, short-meetings) • Establishment of a COVID-19 Hotline for staff, operated by hygiene department, to answer questions regarding current regulations	• Top-down external communication of infection control regulations from responsible institutions via regional network meetings of physicians who passed the information on to nursing staff • Top-down internal communication - regarding COVID-19 isolation unit - regarding external infection control regulations
Staff scheduling	• Day-to-day staff scheduling • Flexibilization of working hours: nurses with children started shift later • On-call duty schedule • 12 h-shifts on weekends for a limited period	• 12 h-shifts
Team work and cooperation	• Interprofessional team work, e.g., through meetings and joint handovers with physicians and physiotherapists • Close intersectional team work within the whole organization • Assignment of one whole team of another unit on the COVID-19 unit for one month (instead of single assigning nurses of different teams) • Team counseling and supervision sessions	• Distribution of patients with COVID-19 across hospitals by emergency service • Cancellation of support by military or trainees
**Interventions perceived as successful**		
Work flow	• Adaptation of shift procedure to new situation (e.g., new tasks) • Flexibilization: decision and assignment of daily (care) tasks in the morning	
Provision of patient care	• Temporary unit closure due to high numbers of COVID-19 patients • Management of patient flow: Centralization of emergency cases in main clinic, patients with mild-infections were transferred to smaller clinic of same organization • Optimization of nursing care through the use of prone positioning kits	
Resident-relative-interaction	• Enabling communication via Smartphone, WhatsApp, Video-Calls (procurement of Tablets etc.)	
**Interventions perceived as less successful**		
Training	• Continuous training of nursing staff (hygiene measures, COVID-19 disease)	

**Table 3:** Frequencies of main category and key implementation factors

	Total		Successful		Less successful	
	*N*	%	*N*	%	*N*	%
**Main category (intervention)**						
Infection control	21	41	13	36	8	53
Staff scheduling	9	18	8	22	1	7
Team work and cooperation	8	16	6	17	2	13
Communication and information flow	5	10	2	6	3	20
Work flow	3	6	3	8	0	0
Provision of care	3	6	3	8	0	0
Resident-relative-interaction	1	2	1	3	0	0
Training	1	2	0	0	1	7
*T* *otal*	51	100	36	100	15	100
**Aim (multiple answers)**						
Infection prevention	15	33	11	31	4	44
Improving operational efficiency	7	16	7	19	0	0
Ensuring implementation of intervention	7	16	3	8	4	44
Reducing staff burden	5	11	4	11	1	11
Ensuring continuity of care	4	9	4	11	0	0
Ensuring patient safety	3	7	3	8	0	0
Enhancing staff resilience	3	7	3	8	0	0
Balancing staff and patient safety priorities	1	2	1	3	0	0
*T* *otal*	45	100	36	100	9	100
**Promoting factor (multiple answers)**						
Team	18	34	18	39	0	0
Facility	15	28	13	28	2	29
Management and leadership	12	23	10	22	2	29
Individual staff-related factors	4	8	2	4	2	29
Work environment and organization	4	8	3	7	1	14
*T* *otal*	53	100	46	100	7	100
**Hindering factor (multiple answers)**						
Work environment and organization	26	54	19	58	7	47
Facility	10	21	5	15	5	33
Individual staff-related factors	7	15	6	18	1	7
Management and leadership	3	6	1	3	2	13
Team	1	2	1	3	0	0
Not existing	1	2	1	3	0	0
*T* *otal*	48	100	33	100	15	100
**Source of challenges (multiple answers)**						
Nursing staff	10	35	8	47	2	17
Infrastructure	9	31	7	41	2	17
Regulations, Information, Communication	6	21	1	6	5	42
Relatives	3	10	0	0	3	25
Management	1	3	1	6	0	0
*T* *otal*	29	100	17	100	12	100
**Challenges (multiple answers)**						
Lack of staff acceptance	5	17	4	24	1	8
Staff shortages	5	17	4	24	1	8
High workload	3	10	0	0	3	23
Inadequate facility layout	2	7	1	6	1	8
Moral distress	2	7	2	12	0	0
Unit closure	2	7	1	6	1	8
Fear of the unfamiliar situation	2	7	2	12	0	0
Ineffective information flow	2	7	0	0	2	15
Lack of external cooperation	1	3	1	6	0	0
Withdrawal of leadership	1	3	1	6	0	0
Time constraints	1	3	1	6	0	0
Psychological distress among residents	1	3	0	0	1	8
Staff management	1	3	0	0	1	8
Testing	1	3	0	0	1	8
Lack of clear guidelines	1	3	0	0	1	8
*Total*	30	100	17	100	13	100
**Idea**						
Management	26	62	20	61	6	67
Multi-stakeholder involvement	9	21	7	21	2	22
External	4	10	3	9	1	11
Team	2	5	2	6	0	0
Hygiene specialists	1	2	1	3	0	0
*T* *otal*	42	100	33	100	9	100

Notes. N = number, % = percent

#### Reasons for positive or negative perceptions of intervention success

General reasons for *positive appraisals of the interventions* are their immediate practicality, the prevention of outbreaks, as well as their positive impact on care delivery and staff well-being. For instance, rapid information flows ensured that everyone was quickly updated. Cohorting of staff improved continuity of care, reduced fluctuation, prevented the infection spread, and strengthened team dynamics as well as resident relationships. Parent-friendly shifts and day-to-day scheduling facilitated staffing during high absence rates. The involvement of a hygiene nurses in pandemic management reduced stress experience of nursing staff. Infection control interventions such as testing, mask-wearing, isolation units, and stockpiling protective equipment, prevented outbreaks. Interprofessional collaboration on equal footing enhanced care quality. Interventions such as prone positioning sets and closing units, optimized time and staff resources. Supportive structures like supervision, feedback rounds, and hotlines helped clarify concerns, reduced psychological strain, and ensured consistent care throughout the pandemic. In the following quote a participant describes why appointing a hygiene officer was perceived as successful:

So the best thing was really our hygiene officer, who took some of the burden off the nursing staff. And it was actually the case that throughout the entire company—even at the district association—they wanted to request our [name of hygiene officer], because, well, that’s where people first became aware of it. Yeah, because it really was something like that; I mean, we used to have one hygiene officer from each team. […] But that had never been implemented professionally the way it is now through our hygiene officer. And so that was the biggest benefit for us. I mean, if a company has a really capable hygiene officer, it’s like winning the lottery. From the very beginning, and ever since then, our hygiene officer and our nursing manager really been dealing with all of this. […] They dealt with all these regional health departments, with all these regulations. Friday afternoon, when the government made another decision. – nurse leader in a rural long-term care facility (LP04WB02)

*Negative appraisals* of *infection control interventions* are attributed to several factors, including regulations on patient testing based on vaccination status, frequent changes in testing protocols, long waiting times for test results, inaccurate test outcomes, and infrastructural limitations such as inadequate or absent waiting areas for patient testing. Regarding hygiene measures, the mandatory use of PPE without mitigating its negative effects on the residents was criticized. Additional reasons for negative perceptions included staff non-compliance, infrastructural constraints (such as no space for testing), and rash decisions on when full protective gear should be worn. Cohorting staff following outbreaks led to conflicts among nursing personnel, and visitation bans were perceived as detrimental to the residents’ health.

*Negative appraisals* of top-down external communication about infection control regulations were often due to information being delivered too quickly, too frequently, and in an unclear or ambiguous manner. A lack of information or the absence of clear guidelines from the hospital management contributed to feelings of abandonment and negatively affected internal communication. Additionally, digital communication and insufficient personnel resources for the translation of external directives into internal guidelines hindered effective communication as well. Overall, managing and organizing the information flows was time-consuming. Extended 12-hour shifts strained staff. Also, the transfer of patients to COVID-19 hospital centers based on bed capacity overwhelmed these facilities due to staffing shortages. The following quote illustrates the complex situation when organizing top-down external communication and internal communication:

*The flow of information is definitely an area where things certainly didn’t go quite as smoothly as they could have. […] we did evaluate it internally afterward. […] As I said*,* the problem really lay in the COVID regulations. There was*,* let’s say*,* a meeting for the region with members in a high management level —once a week*,* always on Mondays*,* Monday afternoons—a large online conference […] where our doctors participated. So they received the latest information on Monday afternoons*,* and we always had a internal task force meeting on Tuesday mornings at 7:30 a.m. So in that regard*,* we really couldn’t have acted any more promptly. […] the staff—I know they wanted to receive more information in print form*,* so that was the feedback we received—but you have to say*,* we were also… There were also plenty of absences - also in the administrative department […] the dissemination of information was really a problem. Certainly via the intranet platform—optimizing that is definitely a possibility. […] also ensuring that the flow of information reaches every nurse within the unit. That’s probably another challenge*,* because with all the constant changes and the heavy workload*,* nurses sometimes just don’t have the time or the patience to look things up online right now. That’s why they said they’d prefer it on paper*,* with the most important information listed there*,* so we can quickly review it during the handover. Because computer workstations are limited*,* by the time they actually get to a computer*,* they have to handle medical and nursing documentation on the computer and simply don’t have the time or energy to look up information online. – Director of Nursing of a non-metropolitan hospital (KH14PD15)*

#### Leadership hierarchy-level and care setting

Among the positively perceived interventions, 21 were reported by nurse leaders from the operative management level, and 15 by those from the middle and strategic management level. Of the 15 negatively perceived interventions, eight were mentioned by operative-level leaders and seven by middle and strategic management leaders. Regarding care settings, participants from hospitals reported 20 interventions perceived as successful and six perceived as less successful, while those from long-term care facilities reported 16 interventions with positive appraisal and nine with negative appraisal.

### Question 2: Key implementation factors

Key factors affecting the intervention implementation process included the aim of the intervention, promoting and hindering factors, evolving challenges during the implementation process, the origin of the idea and the source of initiation, the participation of nursing staff, staff acceptance, nurse leader role, and systematic evaluation of the intervention. The total frequencies and based-on leaders’ appraisals are presented in Tables 3 and 4.

**Table 4:** Frequencies of key implementation factors, with correlations to positive intervention appraisal

	Total		Successful		Less successful		Pearson *r* (*p*) (1 = succ.)
	*N*	%	*N*	%	*N*	%	
**Initiation of implementation (1 = internal)**							0.17 (0.34)
External	11	31	7	27	4	44	
Internal	24	69	19	73	5	56	
*T* *otal*	35	100	26	100	9	100	
**Participation of nursing service during implementation (2 = participation)**							0.19 (0.24)
Non-participation	13	31	9	27	4	44	
Preliminary stage of participation	13	31	10	30	3	33	
Yes	16	38	14	42	2	22	
*T* *otal*	42	100	33	100	9	100	
**Role of nurse leader (2 = active: development)**							0.12 (0.47)
Passive	2	5	2	7	0	0	
Active: administration, leadership of staff	31	80	23	74	8	100	
Active: Development	6	15	6	19	0	0	
*T* *otal*	39	100	31	100	8	100	
**Acceptance by nursing staff (2 = low or no acceptance)**							**0**.35* (0.03)
Low or no acceptance	4	10	2	6	2	29	
Declining over time	6	15	4	13	2	29	
Varying	1	3	1	3	0	0	
Increasing over time	5	13	4	13	1	14	
High acceptance	23	59	21	66	2	29	
*T* *otal*	39	100	32	100	7	100	
**Evaluation (1 = systematic)**							-0.13 (0.44)
Leader reflection or no evaluation	32	84	26	87	6	75	
Systematic evaluation	6	16	4	13	2	25	
*T* *otal*	38	100	30	100	8	100	
**Hierarchy-level (2 = strategic)**							0.01 (0.96)
operative	29	57	21	58	8	53	
middle	13	26	8	22	5	33	
strategic	9	18	7	19	2	13	
*T* *otal*	51	100	36	100	15	100	
**Setting (1 = hospital)**							0.14 (0.32)
long-term care	25	49	16	44	9	60	
hospital	26	51	20	56	6	40	
*T* *otal*	51	100	36	44	15	100	

Notes. N = number, % = percent, p = p-value, *p-value < 0.05 (two-tailed)

#### Aim of intervention

Interventions relating to *resident-relative interaction* primarily aimed to ensure patient safety. Interventions addressing *workflow* focused on balancing staff and patient safety priorities, improving operational efficiency, and preventing infection. *Staff scheduling* interventions sought to enhance efficiency, reduce staff burden, and ensure continuity of care. *Teamwork and cooperation* measures aimed improving efficiency, reducing staff burden, ensuring continuity of care, and enhancing staff resilience. *Infection control* interventions targeted infection prevention or reducing staff burden. *Communication and information* flow interventions aimed to facilitate the implementation of other interventions. Finally, interventions regarding *care provision* focused on improving operational efficiency, whereas training interventions were designed to support successful implementation.

#### Promoting and hindering factors during implementation process

Promoting and hindering factors can be categorized according to their source: Team, individual staff-related factors, facility, management and leadership, or work-environment and organization. The majority of statements regarding promoting factors relates to the team, the facility and to management and leadership. The majority of statements on hindering factors is related to work-environment and organization or facility.

*Promoting factors* associated with *positively perceived interventions* at the team level include effective teamwork within nursing staff and trans-sectoral or interprofessional collaboration (e.g., with physicians). Individual staff-related factors highlighted by the participants encompassed creativity, motivation, engagement, situational understanding, and pragmatism. At the facility level, key promoting factors were the provision of essential materials (often facilitated through cooperation), the availability of testing capacities and personnel, the deployment of experts (such as hygiene specialists), an infrastructure enabling the separation of infected residents from non-infected ones, a culture of learning from errors, and flat hierarchical structures. Regarding management and leadership, facilitating factors include clear communication and information flow across hierarchical levels (e.g., brief additional meetings), managerial support for nurse leaders, honest dialogue with staff, recognition of nurses´ contributions, and the provision of incentives. In terms of work environment and organization, factors such as residents’ understanding for the pandemic situation, flexible and family-friendly scheduling, streamlined communication, patient testing in emergency units prior to admission, and on-duty scheduling were emphasized.

Interestingly, *promoting factors* were also reported in relation to *negatively perceived interventions*. These included individual creativity and pragmatism, expert deployment and a culture of learning from errors at the facility level, honest and transparent communication and leadership attentive to staff preferences at the management level, and the use of intranet for rapid information dissemination alongside cooperative residents within the organizational environment.

*Hindering factors* identified for *positively perceived interventions* encompass team conflicts arising from perceived unfair task distribution, absence of an assigned unit physician, physicians’ fear or poor cooperation with physicians. Individual-level obstacles included long-term staff absences, limited shift availability, physical burden from mask-wearing, psychological strain, fear, and moral distress. Facility-related barriers referred to infrastructural limitations (e.g., lack of isolation rooms), staff shortages linked to mandatory vaccination, and financial difficulties due to changing patient loads. Lack of support from the facility management was mentioned as an aspect of leadership challenges. Organizational hindrances comprised the closure of schools and kindergartens, diversion of staff capacity to interventions at the expense of other tasks, disorganized hygiene material management, delays and inaccuracies in test results, conflicts with residents’ relatives (e.g., regarding the necessity to wear face masks), reduced face-to-face communication, rapidly changing external regulations, absence of external services for residents, poor inter-institutional communication (e.g., between health departments), and decreased social participation for residents.

Similarly, *hindering factors* for *negatively perceived interventions* included challenges in balancing diverse staff needs and preferences, physical strain due to PPE usage, prolonged test result turnaround times resulting in overcrowded emergency units, infrastructural deficits (e.g., insufficient testing spaces and cafeterias), restricted intranet access due to limited computer availability, and infection-related staff shortages. Management-related barriers encompass halted recruitment of trainees and military personnel, a lack of managerial support, and dissatisfaction with leadership in the facility. Organizational issues involve increased staff workload, controversial vaccination policies, extended overtime, resident frustration due to prolonged hygiene measures, and communication difficulties with residents.

#### Challenges during the implementation process

The main source of challenges during the intervention implementation are related to the theme nursing staff (eight successful, two less successful), followed by infrastructural restrictions (seven successful, two less successful). Other sources are relatives (zero successful, three less successful), regulations and guidelines (one successful, three less successful), and management (one successful, two less successful).

Challenges were multifaceted and can be summarized as follows. Across both positive and negative participants’ appraisals, common challenges included the lack of staff’s acceptance of the intervention, inadequate facility layout, staff shortages, and unit closures. Challenges unique to positively perceived interventions included lack of external cooperation, moral distress, fear of unfamiliar situations, withdrawal of nurse leaders, and time constraints. Conversely, negatively perceived interventions were characterized by psychological distress among residents, difficulties in staff management, high workload, issues with testing (e.g., time constraints, no material, no testing rooms, no personnel, false results, waiting time on results), ineffective information flow, and a lack of clear guidelines. In the following quote, a participant explains how changes in work organization helped mitigate infrastructural challenges implementing the intervention “spatial separation of residential area due to infection control”.

*Well, we couldn’t change the rooms themselves. We just adjusted our work procedures—for example, we stopped doing shift handoffs in the staff room. Items needed in both areas that were previously kept in the staff room were now stored centrally. – Manager of nursing service in a rural long-term care facility* (LP02PDL+HL03)

### Origin of idea

The majority of intervention ideas originated from the management (20 successful, six less successful). Additional sources included nursing teams, hygiene specialists, external stakeholders, and initiatives developed through collaborative processes involving both multiple actors.

Occasions for intervention implementation covered a broad range of topics. The most frequently cited occasion was the epidemiological situation (*n* = 6), followed by initiatives driven by operational-level leadership (*n* = 4). Other occasions included the need to comply with public health regulations, staffing or patient safety considerations, the emergence of new information or knowledge-sharing efforts, process optimization, responses to internal outbreaks, and adherence to hospital policies.

Of the interventions reported, 24 (19 successful, five less successful) were initiated internally within the facility, while eleven (seven successful, four less successful) were initiated externally.

### Participation of nursing staff

In 16 interventions (14 successful, two less successful), nursing staff were reported to have actively participated in at least one stage of the implementation process such as contributing to decision-making or the design of hygiene protocols and staff scheduling. In 13 interventions (ten successful, three less successful), a more preliminary form of participation was noted, including activities such as sharing information or being informed about the intervention. In contrast, 13 interventions (nine successful, four less successful) involved no reported participation by nursing staff, with implementation characterized by the execution of managerial directives.

#### Staff acceptance

High acceptance of the intervention by nursing staff was reported in 23 cases (21 successful, two less successful). In contrast, low or no acceptance was reported in four interventions (two successful, two less successful). For six interventions (four successful, two less successful), a decline in acceptance over time was reported, while in one intervention perceived as successful, acceptance increased over time. In five interventions (four successful, one less successful), acceptance varied among staff or depended on specific components of the intervention.

#### Nurse leader role

In six positively and zero negatively perceived interventions, the interviewed nurse leader played an active role during the development phase of the intervention. In 31 interventions (23 successful, eight less successful), the nurse leader’s role primarily involved administering the intervention or instructing nursing staff. In two positively perceived interventions (0 negative), the nurse leader assumed a passive role.

#### Evaluation

Systematic evaluation of the intervention was reported in six cases (four successful, two less successful). For the majority of interventions (*n* = 32; 26 successful, six less successful), no formal evaluation or only informal reflection by the nurse leader on intervention effectiveness was reported.

### Question 3: Correlations between implementation factors and positive intervention appraisal

Correlation with intervention appraisal of success or failure were reported for source of initiation, participation of nursing service, staff acceptance, nurse leader role, evaluation, leadership hierarchy level and care setting (Table 4). The presented correlation between quantitized themes and corresponding categories with intervention appraisal are presented as exploratory findings, derived from qualitative data.

A moderate and statistically significant positive correlation was found between nursing staff acceptance of the intervention and its positive appraisal by nurse leaders (r(37) = 0.35, *p* = 0.03). All other examined relationships showed either weak or very weak correlations and did not reach statistical significance. These included correlations between positive appraisal and internal initiation (r(33) = 0.17, *p* = 0.34), nursing staff participation (r(40) = 0.19, *p* = 0.24), active leadership role (r(37) = 0.12, *p* = 0.47), systematic evaluation (r(36) = − 0.13, *p* = 0.44), management hierarchy level (r(49) = 0.01, *p* = 0.96), and hospital setting (r(49) = 0.14, *p* = 0.32).

## Discussion

This study explored nurse leaders’ perspectives on the success of work-organizational interventions implemented in inpatient care facilities during the COVID-19 pandemic across hospitals and long-term care settings in Saxony, Germany. Through retrospective qualitative interviews, we sought to understand, first, which interventions were perceived as successful or less successful, second, to identify key factors influencing their implementation, and, third, to explore how these factors correlate with nurse leaders’ appraisals. Although this approach provides the advantage of capturing in-depth insights into contextual and practical factors that shaped implementation, the findings reflect nurse leaders’ perceptions of the reported interventions rather than an objective assessment of interventions’ effectiveness.

### Question 1: Main themes and interventions

Regarding the first research question, nurse leaders reported that over 70% of the 51 interventions were perceived as successful. Positive appraisal received those measures relating to workflow, staff scheduling, resident-relative interaction, and care provision, while interventions addressing communication, teamwork, and infection control elicited more mixed views. Interestingly, according to nurse leaders’ perceptions, infection control interventions received both the highest number of positive and negative appraisals. For example, testing and cohorting of staff were both perceived as successful and less successful. Our study confirms and extends prior empirical findings related to pandemic-induced organizational change in nursing. Similar to reports by Diehl et al. [8] and Nicolakakis et al. [9], interventions addressing infection control measures (e.g., spatial reorganization, PPE protocols) received mixed perceptions, but were perceived as necessary but challenging due to rapidly shifting regulations and resource constraints. The mixed perceptions regarding interventions aimed at communication and teamwork reflect ongoing difficulties managing psychosocial risks under crisis conditions [3, 10]. In conclusion, it underscores the assumption that nuances in implementation process are key for perceived interventions success.

### Question 2: Key implementation factors

With regard to the second question, facilitators such as effective teamwork and interprofessional cooperation, motivated staff, leadership support, and clear communication were identified. Notably, some factors, such as creativity and pragmatism, deployment of experts, culture of learning from errors, honest and transparent communication, cooperative residents, leadership attentive to staff, were reported as both facilitators in both negative and positive perceptions of success. Moreover, some hindrances, such as long test results waiting time, physical and psychological stress, negative impact on residents, internal and external communication difficulties and regulatory complexity, lack of support by leadership, infrastructural restrictions, staff shortages, were reported in both negative and positive perceptions of success. This underscores the general importance of the presence or absence of these factors in the implementation process.

Moreover, promoting and hindering factors as well as implementation challenges consistently mapped to core domains, such as team dynamics, individual staff resources, leadership and management, communication, infrastructural conditions, and broader organizational systems. These overlapping dynamics suggest that key difference lay not in presence or absence of mentioned factors but in structural and interpersonal supportive conditions enabling nurses and their leaders to effectively manage the pandemic situation.

Our findings align with literature emphasizing the multifaceted nature of pandemic challenges in healthcare [21, 35]. These findings highlight that interventions must be context-sensitive, flexible, and responsive to dynamic organizational and individual needs. It also highlights the unreadiness of care facilities infrastructure for a crisis situation. Future facility renovations or new constructions should develop or consider solutions to enable infrastructural flexibility to adapt to crisis management needs. It further emphasizes the ongoing shortage of nursing personnel prior to the pandemic, which hindered the implementation of interventions regardless of their overall appraisal.

### Question 3: Correlations between implementation factors and positive intervention appraisal

In response to the third research question, our results suggest a significant positive correlation between nursing staff acceptance and positive intervention perception. However, other factors such as nursing staff participation, nurse leaders’ active roles, source of intervention initiation, and formal evaluation showed weaker and non-significant associations with perceived success. These correlations are exploratory and should be interpreted with caution, as they are based on variables derived from qualitative data. Still, our results underscore the central role of staff acceptance in shaping the perceived success of work-organizational interventions, which is consistent with Nielsen and Randall [25] process evaluation framework. Interventions that aimed at redesigning nurses work were more likely to be perceived as successful, underscoring the importance of involving nursing staff in the design and adaptation of work processes during crises [26, 27].

Furthermore, much of the prior intervention research and corresponding recommendations have been developed in non-crisis and more stable healthcare contexts. These frameworks assume a relatively controlled environment where interventions can be carefully planned, piloted, and gradually implemented with extensive staff involvement. However, the COVID-19 pandemic presented a highly dynamic, uncertain, and resource-constrained situation, requiring rapid organizational adaptations and often top-down decision-making. Our findings suggest that even in such crisis contexts, staff acceptance may remain a critical determinant of intervention success, but the pathways to achieving this acceptance may differ. During the pandemic, continuity of care, nurses’ immediate practical needs, perceived relevance, and feasibility of interventions became foundational, balancing these elements a question of resources (e.g., time, staff). This shifts the focus from traditional participatory approaches—which may be difficult to implement fully under crisis conditions—to a more pragmatic approach where transparent communication, leadership responsiveness to urgent staff concerns, and individual and facility adaptability to rapidly changing circumstances may be more relevant to fostering acceptance.

Interestingly, other theoretically important factors—such as nurse leaders’ active engagement, formal evaluation of interventions, and the origin of the intervention (top-down vs. bottom-up)—showed weak or no significant association with perceived success. This may indicate that during the acute, high-pressure context of a pandemic, nursing acceptance outweighs formal leadership-driven processes or evaluation practices. These findings support previous research emphasizing that in rapidly evolving situations, practical feasibility and staff acceptance can be more critical than formalized strategies or leadership involvement alone [31, 32].

Finally, when interpreting the results, it must be considered, that organizational factors likely influenced several of the findings reported in this study. For example, the structured and hierarchical nature of the German healthcare system, combined with strong regulatory and procedural orientation, especially during national pandemic management, may have shaped nurse leaders’ experiences and perceptions, particularly in relation to decision-making, communication, and implementation of pandemic-related measures. Differences in resources, staffing levels, and management practices across healthcare facilities and ownership type may also have contributed to variability in responses.

### Strength and limitations

This study’s exploratory sequential mixed-methods design provides in-depth insights but also presents several limitations. First, the retrospective nature of data collection may have introduced recall bias, as participants reflected on past events during a highly stressful and rapidly evolving pandemic situation. Second, the study was restricted to nurse leaders from healthcare facilities in Saxony, Germany, limiting the transferability of results to other regions or healthcare systems with different pandemic responses or organizational cultures. The German healthcare system, for example, is characterized by a structured, hierarchical culture with strong regulatory and procedural orientation. Third, the perspectives of frontline nursing staff were not included, which constrains the comprehensiveness of understanding intervention success. Nurse leaders’ views, while valuable, may differ from those of nursing staff who directly experienced the interventions’ impact. Still, in our sample we included nurse leaders on operational-level, which are every day in close contact with nursing staff. Furthermore, reported questions were part of a broader study. Therefore, full data saturation for the questions analyzed in this paper may not have been achieved. Still, the data collected provided sufficient insight to identify key findings. Finally, reported correlational analyses are exploratory and should be interpreted with caution, due to the transformation of qualitative data into quantitative variables. The limited sample size and instances of missing data due to interview quality limited the statistical power of quantitative analyses, resulting in no significant associations for several theoretically relevant factors. This reduces the generalizability of the reported correlations.

Future research should address these limitations by including larger, more (regionally) diverse samples, integrating nurses’ perspectives, and employing longitudinal mixed-methods approaches across varied organizational cultural and healthcare settings, which can be immediately applied when or shortly after crisis occurred.

### Implication for practice

Our findings underscore that ensuring high nursing staff acceptance must be a key objective when designing and implementing interventions during health crises. This acceptance is best fostered through inclusive communication strategies, participatory decision-making processes, where possible, and the adaptation of interventions to align with the practical realities faced by nursing staff. While nurse leaders hold a crucial mediating role between senior management and employees on operational level, their active involvement alone does not guarantee intervention success unless it effectively translates into genuine staff engagement.

Moreover, although formal evaluation of interventions remains vital for long-term organizational learning, reports of formal evaluation are low. This suggests a need for pragmatic, streamlined evaluation methods that can be feasibly applied even in crisis contexts. Embedding real-time feedback mechanisms and enabling iterative adjustments during implementation can enhance responsiveness and ensure interventions remain relevant and effective under rapidly changing conditions.

For healthcare organizations, this highlights the importance of flexible, context-sensitive implementation strategies that prioritize frontline feedback and foster strong communication channels. Leadership development initiatives should focus on equipping nurse leaders with skills not only in decision-making but also in empowering and motivating staff amidst uncertainty and pressure. For instance, crisis management training for nurse leaders could provide them with a certainty of action and strengthen their resilience in such situations. Furthermore, appointing and educating crisis management experts within the unit teams could possibly reduce the nurse leaders’ workload and increase the nursing staffs’ compliance since they are communicating with educated peers. Additionally, addressing structural challenges such as staffing shortages, infrastructural constraints, and psychosocial risks is essential to create an environment that allows for successful organizational change.

From a public health perspective, these insights emphasize the need for comprehensive crisis preparedness plans that integrate mechanisms for nurses’ involvement and feedback. These should be accompanied by policies that strengthen workforce capacity, workforce resilience and resource availability as well as infrastructural flexibility. This holistic approach is critical to sustaining high-quality care delivery and ensuring the health and wellbeing of nursing staff during future pandemics or other health crises.

## Conclusions

This study provides valuable insights into nurse leaders’ perspectives on successful interventions and key factors for implementation of work-organizational interventions implemented during the COVID-19 pandemic in inpatient care settings. We identified mixed perceptions on similar interventions, suggesting that nuances in implementation process are key for perceived interventions success. Findings on key factors including promoting and hindering factors as well as challenges to successful implementation suggest that addressing structural and psychosocial barriers, strengthening workforce capacities, and actively involving nurses are crucial for perceived intervention success. The limited use of formal intervention evaluation calls for more pragmatic and real-time assessment strategies that can be integrated into crisis responses. Our exploratory correlational findings based on nurse leaders’ responses during interviews suggest that nursing staff acceptance is a relevant determinant of intervention success. While nurse leaders play an important role in mediating between management and nursing staff, fostering genuine staff engagement through transparent communication and flexible, context-sensitive approaches is essential. Our study contributes to improving organizational preparedness and resilience in healthcare. This safeguards both nurses’ wellbeing and patient care quality during ongoing and future health crises.

## Supplementary Information

Below is the link to the electronic supplementary material.

Supplementary Material 1

## Data Availability

Research data within data protection requirements is available upon request. Please contact the corresponding author of this article.

## Declarations

### Use of AI statement

We declare that we prepared this manuscript with the assistance of ChatGPT (OpenAI) for language editing purposes. The authors retained full control over the content and interpretation.
