# Supplementary material for: Nurse leaders’ appraisal of work-organizational interventions during COVID-19: an interview study in German hospitals and long-term care facilities
Source: BMC Nurs. 2026 May 21;25:468. doi: 10.1186/s12912-026-04781-y (PMC13198049; doi:10.1186/s12912-026-04781-y)
Supplement: Supplementary file 1 — Supplementary Material 1 [file 12912_2026_4781_MOESM1_ESM.pdf]

Guideline interview

(1) Interview control sheet

**Date**		**Type of facility**	☐ Hospital<br>☐ Nursing home
**Start**		**Name of the institution**	
**End**		**Location of the facility**	
**Interview format**	☐ Digital<br>☐ Personal, location: ______________________	**Sponsorship**	
**Participant code**		**Professional position of the participant**	□ Director of Nursing □ Nursing service management<br>□ Team management □ Facility management
**Interview code**		**Type of station**	
**Interest in participating in management exchange meetings**		□ Yes □ No □ No statement possible	
Interaction in the interview, difficult passages:		Additional information, special incidents or anomalies in the interview:	
Impression of the motivation to participate:			
receive the required documents:<br>☐ Declaration of consent for participation<br>☐ Declaration of consent to data protection<br>☐ Bank details	Readiness for<br>☐ Video documentation<br>☐ Audio documentation<br>☐ Photo documentation	Short memo: Note important, repetitive or prominent topics	

(2) Greeting

"We at the HTW Dresden and the BAuA are delighted that you have agreed to take part in our interview study as part of the project "Work organization as part of pandemic management in inpatient care"."

(3) Formalities

"Before we start our interview, we first need to clarify a few organizational matters.
You have received the information on study participation and data protection in advance. Do you have any further questions?
I need the signed consent forms for study participation and data protection back.
I also need your bank details for the transfer of the expense allowance. The expense allowance will be transferred by the BAuA to the account you have specified within four weeks. If you have not received the money by then, please get in touch with the BAuA contact person, Maria Zink."

(4) Explanation of the procedure

"I would now like to briefly explain the interview process. The interview is part of a three-year project in which we are interested in what measures can be used to effectively counter the challenges during the COVID-19 pandemic and its consequences in inpatient care facilities.
The pandemic situation in Germany has been very dynamic from the outset and has constantly presented society and care facilities with new situations. These include, for example, the strict contact restrictions during the lockdowns, the varying risk of infection due to the different virus variants or the possibility of vaccination protection as well as the mandatory vaccination of employees in certain healthcare facilities.
Today we would like to look back together on the last two and a half years to reflect the various situations that your organization has faced.
In this interview, I would first like to ask you questions about yourself, followed by questions about your institution's experiences since the start of the pandemic, about your health and specific questions about two strategies or measures of your choice. Finally, we will talk about the lessons you have learned from the pandemic for your institution.

This sentence does not apply to advance shipments:
Only for interviews with Director of Nursing/Nursing service management/Facility management: Our interview consists of two parts. The first part is a short questioning about figures, facts and certain documents of your institution. The second part is the interview.

The interview will last approximately 60 minutes and will be recorded by this audio recorder.
If anything is unclear during the interview, please feel free to ask.
We can take a break at any time if you wish.
Do you still have questions?

Anonymous participant code
Finally, please enter your personal, anonymous participant code. It helps us to anonymize your data.

Enter CODE	Description
________	First letter of the mother's first name (e.g. Maria = M) [If unknown: X]
________	First letter of the father's 1st first name (e.g. Klaus = K) [If unknown: Y]
________	First letter of your own 1st first name (e.g. Petra = P)
________	Day of your own date of birth (e.g. **02**.10.1957 = 02)
________	Day of the mother's date of birth (e.g. **17**.05.1926 = 17)

(5) Start of the guided interview

00. Only for interviews with Director of Nursing/Nursing service management/Facility management **"document analysis"**<br>if not sent in advance: Survey of the document analysis in the form of an interview at this point<br>not applicable if sent in advance:<br>"We sent you a questionnaire in advance with facts, figures and certain documents relating to your facility.<br>Do you have it with you?<br>Did any questions arise when filling out the form?<br>I would take a quick look at the questionnaire and see."
**"If you have no further questions, I would start the recorder and begin the interview."**
• **Start audio recording**

**Part of the interview**	**Interview text**	**Inquiries** (serve to steer the conversation if the interview is slow or as a supplement if there is enough time during the interview)	**Notes for interviewer**
**A.** Socio-demographic questionnaire<br>2 minutes	First of all, I am interested in your person.		Fill in next page together with participant

Participant code _______________ Interview code_______________

Socio-demographic questionnaire

Questions about yourself and your profession

1. what gender are you?

☐ female
☐ Male
☐ diverse

2. what is your age group?

☐ 18-25 years
☐ 26-35 years
☐ 36-45 years
☐ 46- 55 years
☐ 56 to 65 years
☐ >65 years

3. what is your highest educational qualification?

☐ no training qualification
☐ Completion of vocational training in a company (apprenticeship)
☐ Completion of vocational training (vocational or commercial school)
☐ Graduation from a technical college, master craftsman or technical school, vocational or technical academy
☐ University of Applied Sciences degree
☐ University degree
☐ another degree

4 How long have you been working in nursing care?

____

5 How long have you been in your current position?

____

6. how long have you been working in this institution?

____

To be entered by interviewer: 7. what is your current function?

____

To be entered by interviewer: 8. What type of institution are you currently working in?

____

Part of the interview	Interview text	Inquiries	Notes for interviewer
**Introduction**	"Before we come to our main concern, I am interested in the current situation in your facility/ward."		
**B.** Introductory question - *questions about the current pandemic situation in the facility*<br><br>2 minutes	Please briefly describe to what extent your facility is currently affected by COVID-19 infections?<br><br>Which topic in relation to the COVID-19 pandemic is currently preoccupying you?	☐ Is there a current case (patients/residents or in the team)?<br>☐ Are there any quarantine cases (patients/residents or in the team)?<br>☐ What is the mood among employees?<br>☐ How is visitor control regulated? (especially for nursing homes)	
**Transition**	"Thank you for your description. Now I'd like to ask you about your experiences during the pandemic."		
**Introduction to the graphic Appendix 2**	"More than two and a half years have already passed since the start of the pandemic.<br>In our project, we are now interested in how the processes and work in your facility or on your ward/residential area and also the activities of the nursing staff have changed since that time. To make it easier for you to find your way around, I have provided the following graphic. It contains important events during the pandemic and will help you to remember them."		
**C.** Questions about experiences since the start of the pandemic			

Participant code _______________ Interview code_______________

<table>
<tr>
<td rowspan="2">Challenge due to the pandemic

5 minutes</td>
<td>Director of Nursing/Nursing service management/Facility management
First of all, I would like you to describe the challenges your organization has faced since the start of the pandemic?</td>
<td>TEAM MANAGEMENT
First of all, I would like you to describe the challenges you have faced in your ward/residential area since the start of the pandemic.</td>
<td rowspan="2">ask if necessary:
☐ Were there increased supply costs? If so, were there any supply problems?</td>
<td rowspan="2">Use graphic

Of particular interest here: Change in the number of people in need of care, amount/intensity of work, etc.</td>
</tr>
<tr>
<td colspan="2">Please use the graphic to classify your description.</td>
</tr>
<tr>
<td>Changes due to the challenges

5 minutes</td>
<td>Director of Nursing/Nursing service management/Facility management
How have work processes changed as a result of the challenges mentioned?</td>
<td>TEAM MANAGEMENT
How have the work processes on your ward/residential area changed as a result of the challenges mentioned?</td>
<td></td>
<td>Changes due to the sudden confrontation with the pandemic are of interest here
➢ Status before strategies/measures</td>
</tr>
<tr>
<td>Management perspective

2 minutes</td>
<td colspan="2">How has your own work changed specifically as a result of the challenges mentioned?

I would be pleased if you could use this chart as a guide when answering the questions so that you can better classify any changes over time.</td>
<td></td>
<td></td>
</tr>
<tr>
<td>Perspective of nursing staff</td>
<td colspan="2">How has the work of caregivers changed specifically as a result of the challenges mentioned?</td>
<td>Ask if necessary:
How can the changes in the caregivers' work be measured?</td>
<td></td>
</tr>
</table>

Participant code _______________ Interview code_______________

2 minutes	I would be pleased if you could use this chart as a guide when answering the questions so that you can better classify any changes over time.		
Strategies<br><br>10 minutes	What **strategies** have been and are being used in your institution to deal with these challenges?<br><br>Please use the graphic to describe the approximate time at which this strategy was used.	Use of the graphic<br><br>Strategy is above all the definition of the future focus of the organization's activities (v. d. Weth & Strohs., 2002):<br>Here, we use the term strategy to describe the definition of general work organization rules. All pandemic management decisions made in the facility (e.g. pandemic-specific hygiene measures) are based on these rules.	
General measures<br><br>5 minutes	What **measures** have been taken **in the area of care** on the basis of the strategies mentioned?<br><br>You are also welcome to use the graphic to describe the approximate time at which this strategy was used.	Use of the graphic<br><br>Explain measures if necessary:<br>Measures translate the broad directions of strategic planning into concrete commitments to action (von der Weth & Strohschneider, 2002)	
Measures in specific areas	I would now like to ask you about **measures taken in specific areas** of work organization in the care sector.<br>If changes have been made<br>☐ in the care system?<br>☐ Care processes<br>☐ in the shift sequence?<br>☐ in the duty roster?<br>☐ in the organization of teamwork?<br>☐ in information flow design?<br>☐ for decision-making and advice?<br>☐ in the caregivers' area of responsibility?	Hand out worksheet 1<br><br>a. Ask the participant to read through the worksheet and talk about the areas in which changes have occurred.<br><br>*b. The participant should answer the following question: What measure(s) have been taken in this area?*<br><br>c. Then check the box.	

15 minutes	☐ in the involvement of nursing staff in medical rounds?<br>☐ in cooperation with other services?<br>☐ in the planning of patient-centered processes?<br>☐ in dealing with patients & protection from violence?<br>☐ in the area of qualifications, further education and training?<br>in occupational health and safety?		
Transition	"I'm interested in how the work processes have changed as a result of these measures."		
Changes due to the measures<br><br>5 minutes	**Director of Nursing/Nursing service management/Facility management**<br>How have the work processes changed as a result of the measures described?<br><br>***TEAM MANAGEMENT***<br>How have the work processes **on your ward/residential area** changed as a result of the measures described?<br><br>Please use the graphic to classify your description.		Use of the graphic
Management perspective<br><br>2 minutes	How has **your own work** changed specifically as a result of the measures mentioned?<br><br>I would be pleased if you could use this chart as a guide when answering the questions so that you can better classify any changes over time.	Ask if necessary:<br>☐ What support did you receive?<br>☐ What kind of support would you like?<br>☐ What would you have needed to be able to perform your management tasks optimally?	Use graphic
Perspective of nursing staff	How has the **work of caregivers** changed specifically as a result of the measures mentioned?	Ask if necessary:<br>How can the changes in the caregivers' work be measured?	Use of the graphic

Participant code _______________ Interview code_______________

2 minutes	I would be pleased if you could use this chart as a guide when answering the questions so that you can better classify any changes over time		
**Transition**	"Thank you for your descriptions. We are now interested in detailed descriptions of selected strategies or measures for dealing with the changes in requirements due to the COVID-19 pandemic."		
**D.** Mention of two targeted strategies or measures<br>20 minutes in total	1) If you think of all the strategies or measures that your facility has implemented, which was the **most successful** in terms of optimizing caregivers' work organization?	2) If you think of all the strategies or measures your facility has implemented, which was the **least successful in** terms of optimizing caregivers' work organization?	
	"I now have a few more questions relating to the strategy/measure you mentioned."	"We now have a few more questions regarding the strategy/measure you mentioned."	
For interviewer	➔ From here on, use the table on "Strategies/measures of the institution" (Annex 3) and then query the following points for each of the two strategies/measures mentioned:		Appendix 3
Development process of the measures	Can you name approximately when the strategy/measure mentioned started or took place?<br><br>What was the reason for and the aim of this strategy/measure?<br><br>Where did the idea for this strategy/measure come from?		
Participation of caregivers	Were the caregivers involved in the development process or implementation of the strategy/measure?<br><br>If yes:		Potential participation opportunities:<br>- Selection of strategies/measures

	At what point in the process did participation take place?<br><br>How did the participation take place?		- Concrete development of strategies/measures<br>- Implementation of strategies/measures<br>manner (levels of participation):<br>Possibility of contributing own ideas vs. asking for opinions etc.
Management perspective	What role did you play in the development and implementation of the strategy/measure?		This question only if this has not already been made clear in the description.
Implementation of the strategy/measure	What were the factors that helped or hindered implementation?<br><br>How were these measures accepted by the caregivers?<br><br>Were there any challenges in gaining acceptance for the measures among caregivers?		→ Ensure that both facilitating and hindering factors are mentioned
Effectiveness of the strategy/measure	Has the effectiveness of the strategy/measure been reviewed?<br>If so, how did you determine whether the measure worked/achieved its objective?		
**Transition**	"Thank you for your answers. Now I will ask you questions about your health during the pandemic."		
**E.** Questions about *health*<br><br>4 minutes	Has your health or well-being changed since the pandemic?<br>If so, what can these changes be attributed to?<br><br>Please use the graphic to support your statements.		Use of the graphic

Load-optimizing measures	Have explicit **measures** been implemented in the facility to reduce the burden on caregivers?<br><br>Ask if necessary, if time permits:<br>☐ Were these accepted by the employees?<br>☐ Were there specific measures for the return to work of nurses with (suspected) SARS-CoV-2 infection or Covid-19 disease?	In this context, we define measures as steps to optimize work design in order to reduce the negative stress and strain on employees caused by the pandemic (e.g. health circles; employee appraisals).	Please note:<br>Measures that already existed before the start of the pandemic are not in focus.<br><br>If necessary, explain how measures are to be understood here.
**Transition**	"Finally, I am also interested in what lessons you are learning from the pandemic at facility level."		
**F.** Lessons from the pandemic<br><br>6 minutes	In **your opinion**, which areas can your organization **prepare well** for a similar crisis situation in the future?<br>For which areas can she **prepare less well**?		These two questions should be asked.<br>due to dissertation Zink
	What **precautions** would you take or have you already taken to prepare for a similar crisis in the future?		
	Germany also experienced heatwaves and very high temperatures this summer.	Above all, these questions are very essential, but unfortunately they have to come at the end, as they are very specific and only in this way can a structure from the general to the specific be maintained in a meaningful way.	
	**Director of Nursing/Nursing service management/Facility management**<br>**Do very high temperatures affect the work of your facility?**<br>If yes:<br>In what way?<br>How is care work affected?<br>What measures does your organization take?<br><br>***TEAM MANAGEMENT***<br>**Do very high temperatures affect work in your ward/residential area?**<br>If yes:<br>In what way?<br>How is care work affected?<br>What measures are you and your team taking?		

	Will newly applied strategies or measures be retained for future work processes **at facility level**?		These questions can be asked if there is enough time.
4 minutes	**In your opinion**, what would be a suitable strategy for an organization to prepare for such a crisis?		
**Acknowledgments**	"That was the last question. We are now at the end of our conversation. I would like to thank you very much for taking the time to participate in our research project and for helping to make work in the care sector safer and healthier under crisis conditions.<br>For my part, I found our conversation very pleasant and informative."		
Comment by participants	Are there any other aspects that have not been addressed but that you consider relevant to better understand the work of nurses under pandemic conditions?		
**End**	"Thank you very much. I will now stop the audio recording and give you a brief overview of the further course of the study."		
■ **Stop audio recording**			

(6) Explanation of the further course

Finally, I would like to briefly explain the next steps of the study. Based on the results of these interviews, a written survey will be designed, which is aimed at you as a manager and your nursing staff and is expected to take place in the first quarter of 2023. The focus will be on the personal state of health and the measures and strategies taken as a result of the pandemic.

From the results of the interviews and the written survey, the BAuA will derive work organization strategies for dealing with future pandemic-like crisis situations. These will be available to inpatient care facilities from the time of publication.

Last but not least, the BAuA is planning a meeting of all managers who took part in the interview study. This is expected to take place after the end of this interview study in the first quarter of 2023. You will be invited by the BAuA. The aim of the meeting is to create space for an exchange on how to deal with the COVID-19 pandemic among managers. We would be delighted if you are interested in attending.

Interviewer's note: Has the interviewee already expressed an interest in participating?<br><br>____

(7) Conclusion of the interview & farewell

Now I have one last question for you:
All of the things we have just discussed were certainly stressful times and situations. Would you like to tell us anything else from your point of view? Have we forgotten to address anything?

Notes Interviewer:

If there are signs of psychological stress as a result of the conversation:

"If you feel burdened by the conversation, I can recommend the help offered by the BGW. It offers COVID-19 crisis counseling by telephone for BGW policyholders:

Corona hotline for BGW member companies
+49 40 20207-1880

Monday - Thursday: 7.30 a.m. - 4 p.m.
Friday: 7.30 a.m. - 2.30 p.m.

The BGW website also provides **tips on how to deal with crises**.
It also offers **crisis coaching for managers and people with responsibility**."
https://www.bgw-online.de/bgw-online-de/themen/gesund-im-betrieb/gesunde-psyche/telefonische-krisenberatung-fuer-bgw-versicherte-18984

OR/AND:

Recommendation of the PENELOPE project of the AOK/TU Dresden:

- Free training courses on workplace health promotion (https://www.aok.de/fk/plus/betriebliche-gesundheit/weitere-inhalte/bgf-in-der-pflege/digitale-praeventionsangebote-fuer-die-pflege-projekt-penelope/)
- Online training to support the health of nursing staff (https://tu-dresden.de/mn/psychologie/penelope)

---**Goodbye**---
